# Kaposi`s sarcoma associated herpesvirus infection among female sex workers and general population women in Shanghai, China: a cross-sectional study

**DOI:** 10.1186/1471-2334-14-58

**Published:** 2014-02-05

**Authors:** Tiejun Zhang, Ying Yang, Feng Yu, Yanping Zhao, Feifei Lin, Veenu Minhas, Charles Wood, Na He

**Affiliations:** 1 Department of Epidemiology, School of Public Health, The Key Laboratory of Public Health Safety of Ministry of Education, Fudan University, Shanghai, China; 2 Minhang District Center for Disease Control and Prevention, Shanghai, China; 3 Nebraska Center for Virology, School of Biological Sciences, University of Nebraska-Lincoln, Lincoln, NE, USA

**Keywords:** Kaposi`s sarcoma associated herpesvirus, Herpes simplex virus, Seroprevalence, Female sex workers, Women

## Abstract

**Background:**

Limited information on epidemiologic patterns of KSHV, with none focusing on heterosexual transmission, is available in mainland China. To clarify this, a cross-sectional study was conducted among a group of female sex workers (FSW) and general population women (GW) in Shanghai, China.

**Methods:**

An anonymous questionnaire interview was administrated among 600 FSW and 600 GW. Blood samples were collected and tested for antibodies to KSHV, HSV-2, HIV, syphilis and HBsAg. Correlates of KSHV and HSV-2 were examined using multiple logistic regression analysis.

**Results:**

None of the study participants were tested positive for HIV. The seroprevalence of KSHV, HSV-2 , HBV and syphilis was 10.0%, 52.2%, 12.3% and 10.5%, respectively for FSW, and was 11.0%, 15.3%, 9.8% and 2.8%, respectively for GW. KSHV seropositivity was not associated with syphilis and HSV-2 infection as well as sexual practices among either FSW or GW. Nevertheless, HSV-2 infection among FSW was independently associated with being ever married (OR = 1.59; 95%CI: 1.04-2.45), >5 years of prostitution (OR = 2.06; 95%CI: 1.16-3.68) and being syphilis positive (OR = 2.65; 95%CI: 1.43-4.93). HSV-2 infection among GW was independently associated with an age of >35 years (OR = 2.29; 95%CI: 1.07-4.93), having had more than 2 sex partners in the prior 12 months (OR = 6.44; 95%CI: 1.67-24.93) and being syphilis positive (OR = 3.94; 95%CI: 1.38-11.23). A gradual increase of prevalence with the prostitution time group was also detected for HSV-2 and syphilis, but not for KSHV.

**Conclusions:**

KSHV is moderately and equivalently prevalent among FSW and GW. Heterosexual contact is not a predominant route for KSHV transmission among Chinese women.

## Background

Kaposi’s sarcoma-associated herpesvirus (KSHV), also known as human herpesvirus 8 (HHV8), a member of the gamma herpesvirus family, is the infectious etiologic agent associated with all forms of Kaposi’s sarcoma (KS) and two lymphoproliferative disorders: primary effusion lymphoma (PEL) and a subset of multicentric Castleman`s disease [1-4]. A number of epidemiological studies have indicated that the KSHV seroprevalence vary considerably by countries and risk groups, but the routes of transmission have yet to be clearly defined [5]. Both horizontal and mother-to-child transmission routes have been reported [6-11]. Nonsexual and mother-to-child transmission routes are believed to be of importance in endemic areas such as Italy, Zambia, Uganda and other African countries. Generally, infection occurs during early childhood leading to accumulation of infection in the population [6,7,9,10,12,13]. Studies have shown that sexual transmission, particularly among homosexual men, may play a major role in transmission in non-endemic areas such as United States and Western Europe [14-18]. Prevalence and incidence of KSHV infection have been found to be associated with the number of sex partners, syphilis infection and hepatitis B infection. However, the possibility of sexual transmission among heterosexual individuals remained controversial. Some reports suggested that heterosexual transmission may contribute to the acquisition of KSHV infections, whereas other reports found that the sexual mode did not play a significant role in KSHV transmission[19-24]. Data from different regions have not been consistent and needs to be further investigated. Since an effective KSHV vaccine is not likely to be available in the near future, elucidating the exact transmission routes, especially the possibility of heterosexual transmission of KSHV is imperative for developing effective strategies to control KSHV spreading.

Currently, There is limited information on the transmission routes for KSHV infection in mainland Chinese populations. Moreover, no data is available for KSHV seroprevalence among sex workers in China, who have been found to be at high risk of sexually transmitted infections (STIs) including HIV. To determine whether heterosexual transmission of KSHV occurs frequently among female sex worker (FSW) is an important public health concern because of the link with HIV, and its potential threat to other populations. FSWs could potentially play a critical bridging role in spreading KSHV from their high-risk sexual partners to low-risk populations, if a heterosexual transmission route of KSHV could be established. Understanding seroprevalence and potential risk factors of KSHV infection is a prerequisite for the prevention strategy for KSHV transmission and the subsequent diseases associated with this important oncogenic infection. Therefore, we conducted a cross-sectional study among a group of FSWs and a group of general population women (GW) in Shanghai, China, to explore the potential of heterosexual transmission of KSHV infection in China. In parallel, well established STIs including syphilis, HBV, HIV, and particularly herpes simplex virus 2 (HSV-2) infections were also examined. If KSHV could be transmitted through heterosexual contacts, we would expect to observe a higher prevalence of KSHV infection as well as higher prevalences of well established STIs such as syphilis and HSV-2 infections among risk-taking FSWs than low risk GW. We believed that FSW, who were engaging in high risk heterosexual practices and were at risk for STIs, will provide a unique setting to gain insights into the possibility of heterosexual transmission of KSHV.

## Methods

### Study participants and sampling

The present study was conducted in Minhang district, Shanghai, China from June to December in 2011. To be eligible for participating in the study, both FSWs and GW must 1) be aged at 18-65 years, 2) have lived in the community for more than 3 months, and 3) be able to provide a written informed consent. Eligible FSWs must also have engaged in commercial sex (i.e., providing sexual service for money) in the past month.

Sample size was estimated based on our prior knowledge that KSHV prevalence is approximately 4.8% among general population. To estimate KSHV prevalence with 95% confidence and margin of error no larger than 0.02, the minimum sample size was calculated as 430 for each group. To avoid loss of statistical power due to potential non-responses, the expected sample size was increased by 30% to 559 and was practically set as 600 for each group. Due to the large size of potential participants in the study site, a two stage sampling method was applied. At the first stage, four out of the total of twelve township communities were randomly selected from Minhang district. At the second stage, a bibliographic list of working venues for FSWs (such as nightclubs, karaoke bars, and hair salons etc.) was obtained as the sampling frame from each of the four communities. Eligible FSWs in randomly selected working venues were recruited until expected samples were finally met. For selection of GW, a list of households on the streets nearby the selected working venues of FSWs was obtained, from which a number of households were randomly selected and eligible GW at these households were recruited to participate in the survey. Finally, 150 FSWs paired with 150 GW were recruited from each of the four communities, reaching a final sample of 1,200 study subjects including 600 FSWs and 600 GW.

Local public health workers visited each of the selected venues or households and invited qualified individuals by face-to-face in these sites to participate in the study. Those who were inform consented to participate in the study were administered with a face-to-face questionnaire interview by the attending public health workers.

### Questionnaire interview

An anonymous questionnaire interview was conducted for all participants. The first part of the questionnaire requesting information about social and demographic characteristics was administered face-to-face by a trained public health worker. For the second part which solicited the more sensitive information about sexual behaviors, the interview was self-administered. The completed anonymous questionnaires were placed in a large box containing other completed questionnaires, reassuring the participants that no one could identify their completed questionnaire. All interviews took place in a private location. A monetary incentive, equivalent to U.S. $5, was given to each participant.

### Blood collection

Venous blood was collected from participants by professional nurses using disposable sterile needles and tubes and then transferred to local laboratory within 4 hours after collection. Serum samples were stored at -80°C for KSHV, HSV-2, HBV, HIV and syphilis testing. Specimens were coded by unique identification numbers and were analyzed without knowledge of the personal identity of the study participants. The study protocol was reviewed and approved by the Institutional Review Board of Fudan University, China. All study participants provided written informed consent.

### Laboratory testing

#### ***HIV testing***

All serum samples were screened for HIV antibody using an enzyme-linked immunosorbent assay (ELISA; Abbott Laboratories), according to the manufacturer’s instructions. Positive samples were confirmed by a western blot assay (HIV BLOT 2.2; Genelabs Diagnostics, Singapore).

#### ***Syphilis testing***

Serum samples were tested using a rapid plasma reagent test (Span Diagnostics Ltd, Surat, India), and results were confirmed by the *Treponema pallidum* hemaglutination test (TPHA, Syphagen TPHA, Biokit, Spain).

#### ***HBV surface antigen (HBsAg) testing***

HBsAg was tested using an ELISA kit (Wantai Biotech Pharmacy Enterprise Co. Beijing, China). The test was performed following the procedures recommended by the manufacturer. All specimens found to have an absorbance level less than the cut-off value were considered negative. All specimens with an absorbance level greater than the cut-off value were rerun in duplicate to confirm their positive status. If the sample was consistently positive, it was identified as being positive. Those that were re-assayed but fell below the cut-off value were considered negative.

#### ***HSV-2 testing***

HSV-2 IgG antibody was tested using an ELISA technique (HerpeSelect 2 ELISA IgG Kit, Focus Technologies, CA, USA). All tests were performed according to the manufacturers' standard protocols. Equivocal samples were retested using another ELISA technique (HerpeSelect 2 ELISA IgG Kit, Euroimmun, Lübeck, Germany). Only 17 (1.4%) samples were recorded as equivocal on the first cycle test, but not equivocal on re-testing. The results of the 17 samples retested were all negative.

#### ***KSHV serology***

An immunofluoresence assay (IFA) was performed to detect the presence of lytic or latent antigen specific antibodies, as previously reported [25]. Briefly, *Spodoptera frugiperda* clone 9 cells infected with baculovirus expressing ORF65 antigen (lytic antigen) or the ORF73 (latent nucleic antigen, LANA) were harvested, fixed, and spotted individually on separate slides for the further sample testing. All serum samples were then tested at 1:40 dilution. Sera from KS patients and healthy individuals who were previously tested seropositive and seronegative, respectively were used as controls. Only those sera scoring positive by both assays were characterized as KSHV seropositive. Each slide was read independently by two experienced laboratory workers. To determine geometric mean titer (GMT) of KSHV antibody, KSHV seropositive subjects were further tested by IFA on serially diluted samples ranging from 1:40 to 1:10240.

All above serological tests were performed by the same two experienced technicians from the key laboratory of the leading institution of this study, according to the manufacturers' standard protocols. Duplicate negative, positive and blank controls were always analyzed in parallel.

### Statistical analysis

Original questionnaires and laboratory testing results were entered and managed in EpiData3.0. All data subsequently transferred to an SPSS database for further management and statistical analysis. The database for FSWs and GW were initially separate but were merged for the further analysis. Frequencies were calculated for categorical variables. Tests of associations between categorical variables were based on the chi-square test or Fisher’s exact test, whichever was appropriate. Non-parametric tests (Mann-Whitney U tests) were used to assess the difference of geometric mean titer (GMT) of KSHV antibodies between different groups. Univariate logistic regression analysis was initially conducted, followed by multivariate analysis with ‘forced entry’ of all variables examined in the univariate analyses into the multivariate regression model. These variables were considered to be potential confounding variables based on our ‘prior knowledge’ about the causal relationship between risk factors or independent variables and seropositivity of antibodies against KSHV or HSV-2 as well as the significance of examined risk factors or independent variables in univariate analyses. Odds ratios (OR) and 95%CIs were calculated and used to determine whether a variable was associated with antibodies against KSHV and HSV2, respectively. All statistical analyses were performed using SPSS software 15.0 (SPSS, Chicago, Illinois, USA) and GraphPad Prism 5.0 (GraphPad, La, Jolla, CA, USA). A two-sided p-value of 0.05 or less was considered statistically significant.

## Results

### Sociodemographic characteristics

Among the 600 FSW, 94.7% were of Han ethnicity, 56% aged between 18-25 years, 43.5% were single, 20.2% were illiterate or received primary school education and 58.2% received middle school education, 41.8% were living alone, 64.8% had monthly income between 2001-4000 Yuan Chinese RMB and 18.3% had more than that. Among the 600 GW, 96% were of Han ethnicity, 56.3% aged more than 25 years, 68.8% were ever married, 12.5% were illiterate or received primary school education and 35.7% received high school or higher education, 10.7% were living alone whereas 60.5% were living with spouse or sex partners, 60.1% had monthly income less than 2000 Yuan Chinese RMB and only 1.4% had more than 4000 Yuan Chinese RMB. FSW and GW were significantly different in terms of age, marital status, education level, living status and monthly income (Table 1).

**Table 1:** Sociodemographic characteristic and prevalence of KSHV, HSV2, syphilis and HBV infections among study participants

	FSW* (n = 600)	GW* (n = 600)	**Total (N = 1200)**
	**No. (%)**	**No. (%)**	**No. (%)**
**Ethnicity (P = 0.274)**			
Han	568 (94.7)	576 (96.0)	1144 (95.3)
Others	32 (5.3)	24 (4.0)	56 (4.7)
**Age (years, P < 0.001)**			
18-25	336 (56.0)	262 (43.7)	598 (49.8)
26-35	196 (32.7)	175 (29.2)	371 (30.9)
>35	68 (11.3)	163 (27.2)	231 (19.2)
**Marital status (P < 0.001)**			
Single	261 (43.5)	187 (31.2)	448 (37.3)
Ever married	339 (56.5)	413 (68.8)	752 (62.7)
**Education (P < 0.001)**			
Primary school or illiterate	123 (20.2)	75 (12.5)	198 (16.3)
Middle school	355 (58.2)	311 (51.8)	666 (55.1)
High school or higher	132 (21.6)	214 (35.7)	346 (28.6)
**Living (P < 0.001)**			
Alone	251 (41.8)	64 (10.7)	315 (26.2)
With spouse or partner	169 (28.2)	363 (60.5)	532 (44.3)
With others	180 (31.0)	173 (28.8)	353 (29.4)
**Monthly income (Yuan RMB, P < 0.001)**			
≤2000	101(16.8)	361 (60.1)	462 (38.5)
2001-4000	389 (64.8)	231 (38.5)	620 (51.7)
>4000	110 (18.3)	8 (1.4)	118 (9.8)
**Age at first sex (years, P < 0.001)**			
<18	185 (30.8)	35 (5.8)	220 (18.3)
≥18	415 (69.2)	565 (94.2)	980(1.7)
**KSHV (P = 0.572)**			
Positive	60 (10.0)	66 (11.0)	126 (10.5)
Negative	540 (90.0)	534 (89.0)	1074 (89.5)
**HSV-2 (P < 0.001)**			
Positive	313 (52.2)	92 (15.3)	405 (33.8)
Negative	287 (47.8)	508 (84.7)	795 (66.2)
**Syphilis (P < 0.001)**			
Positive	63 (10.5)	17 (2.8)	80 (6.7)
Negative	537 (89.5)	583 (97.2)	1120 (93.3)
**HBsAg (P = 0.168)**			
Positive	74 (12.3)	59 (9.8)	133 (11.1)
Negative	526 (87.7)	541 (90.2)	1067 (88.9)

All P-values in the table were generated from chi-squared tests for comparisons between FSW and GW. *FSW = female sex worker; GW = general population women.

### Sexual behaviors

#### ***Sexual behaviors among FSW***

A substantial proportion (30.8%) of FSW had their first sexual experience before 18 years of age. Among them, 273 (45.5%) had been engaged in prostitution for 1-3 years, 160 (26.7%) for 4-5 years and 167 (27.8%) for more than 5 years. In the past 12 months, 212 (35.3%) FSW reported having had no more than ten sex partners, 185 (30.8%) having had 11-20 sex partners, 92 (15.3%) having had 21-50 sex partners and 111 (18.5%) having had more than 50 sex partners. About 49.3% (296/600) of FSW had used condoms consistently in the past 12 months, 47.5% (285/600) had used condoms occasionally and 3.2% (19/600) had never used condoms.

#### ***Sexual behaviors among GW***

Among the 600 GW, 460 (76.7%) were sexually experienced and only 35 (5.8%) had their first sexual experience before 18 years old (Table 1). Among the 460 sexually experienced GW, 422 (91.7%) reported having only had one sex partner in the past 12 months whereas the other 38 (8.3%) reported having had two or more sex partners or multiple sexual partnership. In the past 12 months, 53 (11.5%) of the 460 sexually experienced GW had used condoms consistently, 203 (44.1%) had used condoms occasionally but 204 (44.3%) had never used condoms.

### Seroprevalence of KSHV, HSV-2, HBV and syphilis infections

As shown in Table 1, the prevalence of KSHV, HSV-2, HBV and syphilis was 10%, 52.2%, 12.3% and 10.5%, respectively for FSW, and was 11.0%, 15.3%, 9.8% and 2.8%, respectively for GW. None of the study participants tested positive for HIV. Although FSW and GW were comparable in the prevalence of KSHV and HBV infections, they were significantly different in the prevalence of HSV-2 and syphilis infections with FSW more likely to be infected with HSV-2 and syphilis than GW (Table 1). Furthermore, after adjusting for age, education and marital status using separate multivariate logistic regression analyses, HSV-2 and syphilis infections but not KSHV and HBV infections were also significantly associated with being FSW. Compared with GW, FSW were more likely to be infected with HSV-2 (OR = 7.38, 95%CI: 5.28-10.31) and syphilis (OR = 5.50, 95%CI: 2.97-10.19).

### Associates of KSHV and HSV-2 infections among FSW

**Figure 1:**
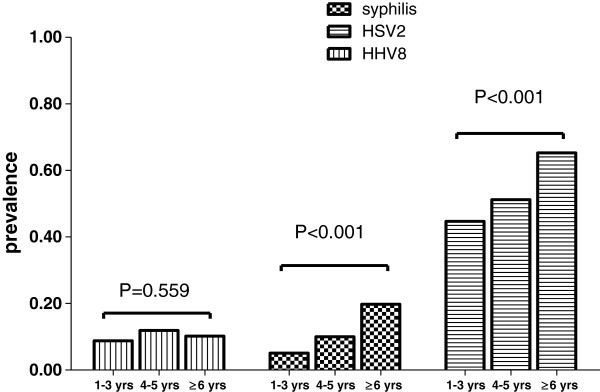
Seroprevalence of KSHV, syphilis and HSV2 among female sex workers according to years of prostitution.

To explore independent associates of KSHV and HSV-2 infections among FSW, two multivariate logistic analyses were performed while adjusting for potential confounding variables. As presented in Table 2, KSHV infection among FSW was not significantly associated with any of the variables listed in the table including those reflecting sexual practices. Nonetheless, HSV-2 infection was significantly associated with marital status, years of prostitution and syphilis infection among FSW. Those who were ever married (OR, 1.59; 95%CI, 1.04-2.45), had been in prostitution for >5 years (OR, 2.06; 95%CI, 1.16-3.68) and were infected with syphilis (OR, 2.65; 95%CI, 1.43-4.93) were more likely to be infected with HSV-2. Furthermore, when analyzed with duration of prostitution, no difference for KSHV seroprevalence was detected across different durations of prostitution (Chi sqaure_*trend*_ =0.326, P = 0.747). However, a positive linear association between HSV-2 seroprevalence and years of prostitution was observed among FSW (Chi sqaure_*trend*_ = 2.922, P < 0.01) (Figure 1).

**Table 2:** Multiple logistic regression analyses of correlates of KSHV and HSV-2 infection among female sex workers (n = 600)

	**KSHV seroprevalence (%)**	**AOR (95%CI)**	**P value**	**HSV2 seroprevalence (%)**	**AOR (95%CI)**	**P value**
**Ethnicity**						
Han	56/568 (9.9)	1.00		297/568 (52.3)	1.00	
Others	4/32 (12.5)	1.42 (0.46-4.37)	0.547	16/32 (50.0)	0.89 (0.42-1.89)	0.760
**Age (years)**						
18-25	33/336 (9.8)	1.00		156/336 (46.4)	1.00	
26-35	21/196 (10.7)	1.09 (0.46-2.56)	0.846	116/196 (59.2)	1.69 (0.77-3.70)	0.185
>35	6/68 (8.8)	0.67 (0.18-2.43)	0.540	41/68 (60.3)	1.44 (0.76-2.72)	0.258
**Marital status**						
Single	27/261 (10.3)	1.00		112/261 (42.9)	1.00	
Ever married	33/339 (9.7)	0.80 (0.39-1.64)	0.539	201/339 (59.3)	1.59 (1.04-2.45)	**0.033**
**Education**						
Primary school or illiterate	13/113 (11.5)	1.00		63/113 (55.8)	1.00	
Middle school	32/335 (9.0)	0.75 (0.36-1.58)	0.457	188/335 (53.0)	0.98 (0.65-1.52)	0.583
High school or higher	15/132 (11.4)	1.01 (0.42-2.49)	0.963	62/132 (47.0)	0.85 (0.48-1.48)	0.960
**Monthly income (Yuan RMB)**						
≤2000	14/101 (13.9)	1.00		64/101 (63.4)	1.00	
2001-4000	33/389 (8.5)	0.48 (0.23-0.97)	0.043	195/389 (50.1)	0.78 (0.43-1.45)	0.448
>4000	13/110 (11.8)	0.81 (0.33-1.97)	0.634	54/110 (49.1)	0.62 (0.37-1.02)	0.060
**Age at first sex (years)**						
<18	19/185 (10.3)	1.00		89/185 (48.1)	1.00	
≥18	41/415 (9.9)	0.89 (0.46-1.69)	0.716	224/415 (54.0)	0.96 (0.64-1.42)	0.822
**Years of prostitution**						
1-3	24/273 (8.8)	1.00		122/273 (44.7)	1.00	
4-5	19/160 (11.9)	1.55 (0.78-3.09)	0.211	82/160 (51.2)	1.22 (0.79-1.86)	0.373
>5	17/167 (10.2)	1.41 (0.56-3.59)	0.468	109/167 (65.3)	2.06 (1.16-3.68)	**0.014**
**Number of sex partners in the past 12 months**						
≤10	19/212 (9.0)	1.00		105/212 (49.5)	1.00	
11-20	17/185 (9.2)	1.05 (0.52-2.16)	0.885	92/185 (49.7)	0.97 (0.64-1.48)	0.908
21-50	10/92 (10.9)	1.37 (0.57-3.27)	0.480	51/92 (55.4)	1.01 (0.59-1.72)	0.970
>50	14/111 (12.6)	1.67 (0.72-3.87)	0.234	65/111 (58.6)	1.05 (0.61-1.80)	0.856
**Condom use in the past 12 months**						
Consistently	28/296 (9.5)	1.00		142/296 (48.0)		
Occasionally	31/285 (10.9)	1.06 (0.58-0.92)	0.834	162/285 (56.8)	1.34 (0.93-1.93)	0.113
Never	1/19 (5.3)	0.52 (0.06-4.14)	0.530	9/19 (47.4)	0.69 (0.26-1.86)	0.463
**HBsAg**						
Negative	52/526 (9.9)	1.00		273/526 (51.9)	1.00	
Positive	8/74 (10.8)	1.26 (0.56-2.87)	0.574	40/74 (54.1)	1.12 (0.67-1.88)	0.664
**Syphilis**						
Negative	56/537 (10.4)	1.00		266/537 (49.5)	1.00	
Positive	4/63 (6.3)	0.51 (0.18-1.51)	0.221	47/63 (74.6)	2.65 (1.43-4.93)	**0.002**
**KSHV**						
Negative	---	---	---	278/540 (51.5)	1.00	
Positive	---	---	---	35/60 (58.3)	1.29 (0.74-2.27)	0.367
**HSV2**						
Negative	25/287 (8.7)	1.00		--	---	---
Positive	35/313 (11.2)	1.32 (0.75-2.32)	0.334	---	---	---

*Note*: OR = odds ratio, CI = confidence interval, which were adjusted for other variables listed in this table by a multiple logistic regression analysis.

### Associates of KSHV and HSV-2 infections among GW

To explore independent associates of KSHV and HSV-2 infections among GW, two multivariate logistic analyses were also performed while adjusting for potential confounding variables. As presented in Table 3, KSHV infection among GW was also not significantly associated with any of the variables listed in the table including those reflecting sexual practices. However, HSV-2 infection was significantly associated with age, number of sex partners in the past 12 months and syphilis infection status among GW. Those who were older than 35 years (OR, 2.29; 95%CI, 1.07-4.93), had had 2 or more sex partners in the past 12 months (OR, 6.44; 95%CI 1.67-24.93) and were infected with syphilis (OR, 3.94; 95%CI 1.38-11.23) were more likely to be infected with HSV-2.

**Table 3:** Multiple logistic regression analyses of correlates of KSHV and HSV2 infection among general population women (n = 600)

	**KSHV seroprevalence (%)**	**AOR (95%CI)**	**P value**	**HSV2 seroprevalence (%)**	**AOR (95%CI)**	**P value**
**Ethnicity**						
Han	63/576 (10.9)	1.00		86/576 (14.9)	1.00	
Other	3/24 (12.5)	1.10 (0.31-3.93)	0.889	6/24 (25.0)	1.70 (0.62-4.66)	0.302
**Age (years)**						
18-25	29/262 (11.1)	1.00		26/262 (9.9)	1.00	
26-35	21/175 (12.0)	1.03 (0.49-2.19)	0.932	27/175 (15.4)	1.21 (0.60-2.45)	0.603
>35	16/163 (9.8)	0.76 (0.31-1.88)	0.551	39/163 (23.9)	2.29 (1.07-4.93)	**0.034**
**Marital status**						
Single	20/187 (10.7)	1.00		17/187 (9.1)	1.00	
Ever married	46/413 (11.1)	1.99 (0.61-6.50)	0.254	75/413 (18.2)	1.18 (0.46-3.05)	0.726
**Education**						
Primary school or illiterate	7/75 (9.3)	1.00		15/75 (20.0)	1.00	
Middle school	31/311 (10.0)	1.07 (0.42-2.72)	0.884	48/311 (15.4)	0.91 (0.39-2.12)	0.834
High school or higher	28/214 (13.1)	1.59 (0.57-4.45)	0.373	29/214 (13.6)	0.96 (0.54-1.68)	0.881
**Monthly income (Yuan RMB)**						
≤2000	39/361 (10.8)	1.00		52/316 (14.4)	1.00	
2001-4000	26/231 (11.3)	1.01 (0.57-1.75)	0.994	37/231 (16.0)	1.11 (0.67-1.83)	0.684
>4000	1/8 (12.5)	1.07 (0.12-10.09)	0.955	3/8 (37.5)	2.03 (0.41-10.13)	0.380
Age at first sex (years)*						
<18	4/35 (11.4)	1.00		5/35 (14.3)	1.00	
≥18	62/565 (11.0)	0.74 (0.24-2.34)	0.613	87/565 (15.4)	1.44 (0.50-4.17)	0.501
Number of sex partners in the past 12 months**						
0	17/140 (12.1)	1.00		9/140 (6.4)	1.00	
1	44/422 (10.4)	0.78 (0.27-2.27)	0.657	71/422 (16.8)	2.54 (0.82-7.94)	0.107
2-5	5/38 (13.2)	1.07 (0.22-5.21)	0.934	12/38 (31.6)	6.44 (1.67-24.93)	**0.007**
**Condom use in the past 12 months**						
Consistently	5/53 (9.4)	1.00		12/53 (22.6)	1.00	
Occasionally	18/203 (8.9)	0.94 (0.32-2.69)	0.899	31/203 (15.3)	0.52 (0.23-1.14)	0.101
Never	43/344 (12.5)	1.69 (0.59-4.86)	0.330	49/344 (14.2)	0.63 (0.28-1.42)	0.266
**HBsAg**						
Negative	59/541 (10.9)	1.00		79/541 (14.6)	1.00	
Positive	7/59 (11.9)	1.06 (0.45-2.49)	0.895	13/59 (22.0)	1.98 (0.98-4.02)	0.055
**Syphilis**						
Negative	65/583 (11.1)	1.00		85/583 (14.6)	1.00	
Positive	1/17 (5.9)	0.50 (0.06-3.97)	0.512	7/17 (41.2)	3.94 (1.38-11.23)	**0.010**
**KSHV**						
Negative	---	---	---	81/534 (15.2)	1.00	
Positive	---	---	---	11/66 (16.7)	1.15 (0.56-2.39)	0.700
**HSV2**						
Negative	55/508 (10.8)	1.00		---	---	---
Positive	11/92 (12.0)	1.17 (0.57-2.41)	0.677	---	---	---

*Note*: OR = odds ratio, CI = confidence interval, which were adjusted for other variables listed in this table by a multiple logistic regression analysis.

*≥ 18 groups including those who never had sex before. ** participants had no sex partnership in prior 12 months including those who never had sex.

### KSHV antibody titers

**Figure 2:**
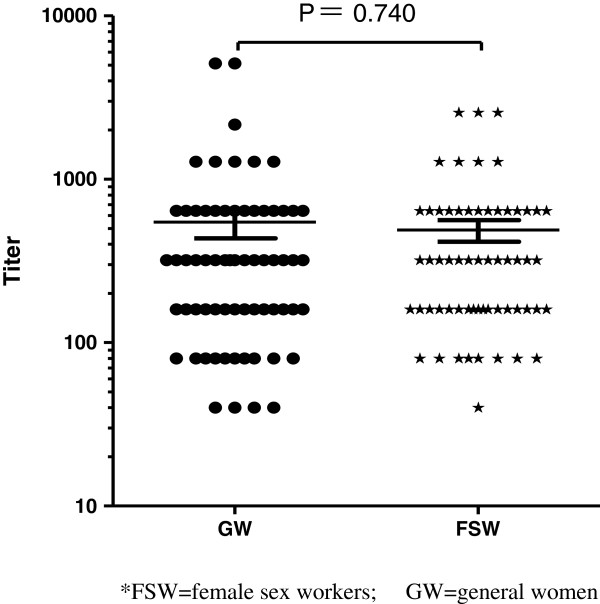
KSHV antibody titer among KSHV seropositive participants.

The GMT of KSHV antibody was 546.7 (95%CI: 323.9-769.4) and 488.7 (95%CI: 340.4-637.0) among KSHV-seropositive FSW and GW, respectively (Figure 2). No significant difference in the GMT of KSHV antibody was observed between these two groups (Mann-Whitney U = 1913, P = 0.740).

## Discussion

This study, to the best of our knowledge, is the first specifically designed epidemiological study of KSHV in Chinese women with a focus on examining the role of heterosexual contacts in KSHV transmission. Given the fact that KSHV is an important opportunistic infection of HIV/AIDS and HIV is clearly associated with STIs and measures of sexual behavior, understanding the KSHV seroepidemiology is of public health importance and contributes to the design of efficient preventive strategies. This study aims to better understand the seroepidemiology and mode of transmission of KSHV in Chinese population.

The moderate KSHV seroprevalence among both FSW and GW identified in this study is comparable to that (11.3%) for the general population but is lower than that (22.2%) for immunocompromised patients and for injection drug users (IDU) (31.2%) in mainland China [26]. It is also much lower than that (32.7%) for Chinese men who have sex with men (MSM) [27], suggesting that unlike male homosexual contacts, heterosexual contacts play limited roles in KSHV transmission in China.

Although previous studies from developed nations reported that KSHV is more common in FSW than in GW [28,29], such observation was not confirmed by our study. The seroprevalence of KSHV was similar between these two groups (10.0% vs. 11.0%). On the contrary, infections with HSV-2 and syphilis [20,30,31], were much more common among FSW than among the GW in our study. Multivariate logistic regression analysis also indicated that being FSW was significantly associated with HSV-2 and syphilis infections but not with KSHV infection. Furthermore, KSHV seropositivity was not associated with syphilis, HSV-2, as well as sexual practices among both FSWs and GW. A significant positive linear association between years of prostitution and HSV-2 seroprevalence was identified among FSWs in this study. Such linear association also did not exist for KSHV infection. The findings suggested ongoing heterosexual transmission of HSV-2 and syphilis during prostitution practices for FSW, whereas KSHV did not follow this mode. These study findings, taken together, suggest that being FSW carries no additional risk for KSHV infection than the GW, and heterosexual contacts are unlikely to play a role in KSHV transmission in our study population. Clearly, the pattern of STIs in FSW suggests high rates of high-risk sexual behavior in this population; however KSHV seropositivity was comparable amongst FSW and GW. This pattern of the lack of association with high-risk sexual behaviour, particularly in FSW and with any markers of STIs strongly suggests that the heterosexual mode does not play a significant role in KSHV transmission in this population.

In the literature, evidence for sexual transmission of KSHV largely comes from studies in homosexual men in developed countries [32-35]. KSHV seropositivity in this population has been consistently found to be associated with markers of sexual activity, such as the number of previous male partners, anal sexual contacts and coinfections with other sexually transmitted agents such as HSV-2 [17,32,35]. Our recently published study, which is the only KSHV seroepidemiologic report among Chinese MSM, also confirmed that KSHV infection was prevalent among this population and was independently associated with receptive anal sex, syphilis and HSV-2 infection [27]. Of note, evidence for the heterosexual transmission of KSHV is less convincing. Although some groups have reported an association of KSHV infection and sexual risk factors [23,24], other studies have shown a lack of evidence for heterosexual transmission [19-22]. In agreement with previous reports from other population, no evidence of heterosexual transmission of KSHV was detected among our study participants. Thus, the inconsistent observations regarding KSHV heterosextual transmission and the consistent observations of high prevalence of KSHV infection among MSM in China and other countries further suggest that unlike homosexual contacts, heterosexual contacts might play less important roles in KSHV transmission, which deserves extensive research.

There are some limitations of the study that need to be considered. First, given that this was a cross-sectional study, our capacity to make valid causal inferences is limited. Second, a relatively low cut-off value of 1.1 for HSV2 assay was used as recommended by the manufacturer, which could potentially overestimate the HSV2 prevalence among the study participants. Third, the present study was conducted among FSWs and GW in a metropolitan area of China, and therefore should be cautiously generalized to the entire Chinese population. Despite these limitations, a strength of our study is the parallel analysis of associates of KSHV and STIs such as HSV-2 infections in both high and low risk study populations. This design could provide an internal control for verifying potential contributions of heterosexual behaviors to KSHV infection. Moreover, the relatively large sample size to ensure the robustness of our results is also one of the strengths of the present study.

## Conclusions

In conclusion, the present study suggests that heterosexual contact is not a predominant route for KSHV transmission among Chinese women. Our observations demonstrate that the precise KSHV transmission routes needs to be further investigated. Future prospective KSHV incidence studies among high risk cohorts to better understand the key determinants of KSHV infection are highly warranted. Such studies would provide important information for the development and implementation of appropriate prevention intervention strategies.

## Competing interests

Author certifies no potential conflicts of interests.

## Authors' contributions

TZ and YY designed the study and drafted the Manuscript. NH and CW oversaw the design and participated in all data analyses, data interpretation and writing the report. FY and YZ were involved in the data collection and interpration. FL and VM participated in study implementation, experiment, data analysis and interpretation. All authors contributed to the preparation of the paper and approved the final version. The corresponding author had full access to all data in the study and final responsibility for the preparation and submission of the results for publication. All authors read and approved the final manuscript.

## Pre-publication history

The pre-publication history for this paper can be accessed here:

http://www.biomedcentral.com/1471-2334/14/58/prepub
